# A scaling law for random walks on networks

**DOI:** 10.1038/ncomms6121

**Published:** 2014-10-14

**Authors:** Theodore J. Perkins, Eric Foxall, Leon Glass, Roderick Edwards

**Affiliations:** 1 Ottawa Hospital Research Institute, 501 Smyth Road, Ottawa, Ontario, Canada K1H 8L6; 2 Department of Mathematics and Statistics, University of Victoria, P. O. Box 1700 STN CSC, Victoria, British Columbia, Canada V8W 2Y2; 3 Department of Physiology, McGill University, 3655 Promenade Sir William Osler, Montreal, Quebec, Canada H3G 1Y6

## Abstract

The dynamics of many natural and artificial systems are well described as random walks on a network: the stochastic behaviour of molecules, traffic patterns on the internet, fluctuations in stock prices and so on. The vast literature on random walks provides many tools for computing properties such as steady-state probabilities or expected hitting times. Previously, however, there has been no general theory describing the distribution of possible paths followed by a random walk. Here, we show that for any random walk on a finite network, there are precisely three mutually exclusive possibilities for the form of the path distribution: finite, stretched exponential and power law. The form of the distribution depends only on the structure of the network, while the stepping probabilities control the parameters of the distribution. We use our theory to explain path distributions in domains such as sports, music, nonlinear dynamics and stochastic chemical kinetics.

## Abstract (web summary)

Random walks on a network describe the dynamics of many natural and artificial systems. Here, Perkins *et al.* study the path distribution—characterizing how the walker moves—and find that it is either finite, stretched exponential or power law for any random walk on a finite network.

## Graphical abstract

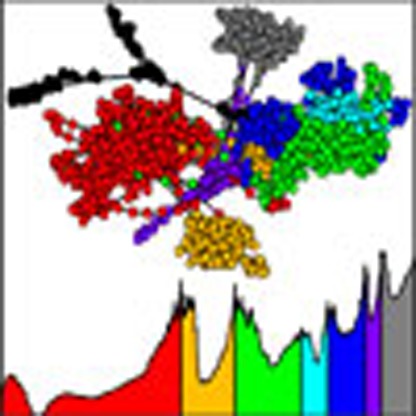

The dynamics of many natural and artificial systems are well described as random walks on a network: protein folding^1^,^2^,^3^, the motions of molecules in rarified gases^4^, information flow in social networks^5^,^6^, traffic and mobility patterns^7^,^8^ and the behaviour of stochastic search algorithms^9^,^10^, to name a few. In the past decade, there has been considerable progress in characterizing first passage times, or the amount of time it takes a random walker to reach a target^11^,^12^,^13^. In contrast, work on characterizing the probability distribution over possible paths has been limited to special types of walks^14^,^15^,^16^,^17^. The path distribution is important because it describes how the walker moves and not just when it arrives. While previous work has largely emphasized the possibility of power law path distributions, other distributions are possible as well.

To see that different types of path distributions may arise from random walks on networks, consider the three networks shown in Fig. 1a–c. Walk A allows only four paths from start node S to end node E. The other networks, which allow a walk to loop back to a node that it has visited before, allow for infinitely many possible paths. For walks B and C, longer paths generally have a lower probability than shorter ones, but there is no strict relationship between path length and path probability, because different steps occur with different probabilities. Suppose we rank the paths in order of decreasing probability, *P*_1_, *P*_2_, *P*_3_,..., where *P*_*r*_ is the probability of the *r*^*th*^ most probable path. Figure 1d shows how the probabilities *P*_*r*_ relate to the ranks *r* for the three walks. For walk C, the relationship is approximately linear on the log–log plot, implying that the path distribution is approximately power law: log *P*_*r*_≈*a*+*b* log *r* or *P*_*r*_≈*cr*^*b*^. However, for walk B, the relationship is clearly curvilinear on the log–log plot, inconsistent with a power law path probability distribution. Instead, the approximately linear relationship between the logarithm of *P*_*r*_ and the square root of *r* for walk B (Fig. 1e) indicates a stretched exponential path distribution: 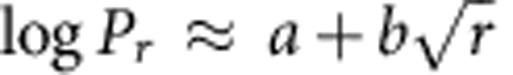, or 
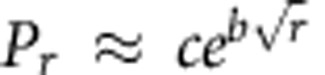
.

Why are the path distributions of these walks so different? Are there other possibilities for the form of the path distribution? How do the form and parameters of the path distribution depend on the structure and transition probabilities of the walk? To date, understanding of these questions has been limited. In the special case of a uniform, memoryless random walk, where each step is equally likely to arrive at any node of the network, the path distribution is known to be power law with *P*_*r*_≈*cr*^−log(*N*)/log(*N*−1)^ for an *N*-node network^14^,^15^,^16^,^18^,^19^. This fact first arose in discussions of Zipf’s law for natural language^20^, although the relevance of random walk models to human language remains a point of contention^21^. Mandelbrot^14^ also argued that, under certain conditions, power law scaling holds for correlated symbol sequences—or, equivalently, random walks on networks, or Markov chains. Still, this left open the questions of whether other types of scaling are possible, and how one might compute the scaling parameters for a given walk.

Here, we state a new scaling law that characterizes the path distribution of any possible random walk on a finite network. We find that there are only three possible forms for the path distribution: finite, stretched exponential and power law. The form of the path distribution depends only on the structure of the network on which the walk takes place, and not on the details of the stepping probabilities. Those probabilities, however, affect the parameters of the distribution. We then use this law to predict path distributions in a variety of domains, finding that both the form and parameters of the empirical path distributions are well explained by our theory.

## Results

### A scaling law for walks on finite networks

Our central result is that if we consider any random walk on a finite network, beginning at a designated start node, ending when it reaches a designated end node (if ever), and if we let *P*_*r*_ denote the probability of the *r*^th^ most probable path from start to end, with ties broken arbitrarily, then there are only three, easily distinguished possibilities for the path probability distribution (see Supplementary Note 1 and ref. 22 for justification):


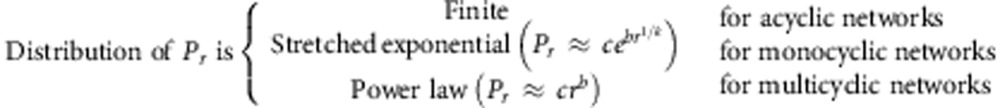


In our categorization, an acyclic network means that there is no path from a live node back to itself, where a live node is one that is reachable from the start node and from which the end node is reachable. We permit cycles (loops) in the non-live part of the network, if any, although these obviously cannot contribute to the path distribution. A monocyclic network has at least one live node participating in a cycle in the network, but no live nodes participating in more than one cycle. In a multicyclic network, at least one live node participates in multiple cycles. Equivalently, the three cases can be discriminated based on the largest eigenvalue *λ*_1_ of the adjacency matrix among the live network nodes, which is less than, equal to, or greater than one, for the acyclic, monocyclic and multicyclic cases, respectively. In the monocyclic case, the parameter *k* equals the maximum number of distinct cycles that may be visited on any path from start to end. In Supplementary Note 2, we describe how to compute the parameter *b*, which is the asymptotic slope of the points on a log *P*_*r*_ versus *r*^1/*k*^ plot for monocyclic networks, or a log *P*_*r*_ versus log *r* plot for multicyclic networks. Despite numerous observations that power law distributions often have *b* near to −1 (ref. 17), one can construct monocyclic walks with any value of *b*<0 and multicyclic walks with any value of *b*<−1 (Supplementary Note 3). Nor is there any necessary connection between the form or parameters of the path distribution and other well-known random walk parameters, such as first passage times or mixing times (Supplementary Note 3). Rather, the path distribution provides a distinct and complementary characterization of the random walk.

### Examples of stretched exponential scaling

**Figure 2:**
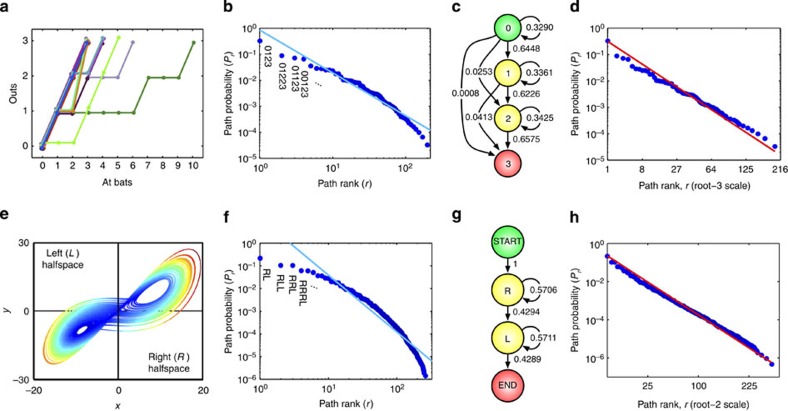
Stretched exponential path distributions explained by random walks on networks: sequences of outs in American baseball and symbolic dynamics of the Lorenz attractor. (**a**) Outs sequences from the half-innings in the first game of the 2012 season between the Kansas City Athletics and the Anaheim Angels (coordinates perturbed slightly for visibility). (**b**) The empirical frequencies of outs sequences from all 2012 Major League baseball games (blue) do not conform to a power law, as shown by the poor fit of a least-squares regression line (cyan). (**c**) A random walk model, with stepping probabilities estimated from the same 2012 data. (**d**) The empirical path probabilities (blue) scale as the third root of rank, with slope close to that predicted by our theory (red). (**e**) *xy* projection of a trajectory of the Lorenz system. Any trajectory can be divided into return paths to the plane *x*=0 travelling in the 
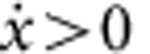
 direction. Qualitatively, each path comprises one or more loops in the right halfspace (*R*, or *x*>0), followed by one or more loops in the left halfspace (*L*, or *x*<0). (**f**) The empirical frequencies of different qualitative paths in a very long simulated trajectory (blue) are not power law. (**g**) A random walk model of the qualitative dynamics with stepping probabilities estimated from the simulated trajectory. (**h**) The empirical frequencies scale as the square root of rank, with slope very close to that predicted by our theory (red).

To demonstrate the use of our theory in understanding path distributions in real systems, and in particular the largely overlooked case of stretched exponential scaling, we turn first to the game of American baseball. Each baseball game has nine innings, and each inning has two halves: one in which the visiting team is ‘at-bat’ and the home team is in the field, and one in which the home team is at-bat and the visiting team is in the field. Each half-inning begins with the batting team at zero ‘outs’ and concludes when the team reaches three outs. Each time an individual player comes up to bat, his actions, and the actions of the players on the field, result in zero or more outs. For instance, if the first batter generated an out, the second batter did not and the third batter generated two outs, as happened twice during the first 2012-season game between the Kansas City Athletics and the Anaheim Angels, then the sequence of total outs would be 0113 (see Fig. 2a for this and other example trajectories). Reasoning about outs sequences is part of the strategy of the game, including the order in which players are selected to bat and the batting instructions they receive. Thus understanding outs sequences in strategically important.

We analysed the observed outs sequences in all 2012-season Major League games available on http://www.retrosheet.org, comprising a total of 30,602 half-innings of baseball, or roughly 1,700 games. We counted the empirical frequencies of different outs sequences and found that they are not consistent with power law scaling (Fig. 2b). We then investigated whether a random walk model could explain the outs sequence distribution. Because each at-bat either leaves the number of outs the same or increases it up to a maximum of three, we chose a walk structured as shown in Fig. 2c. We estimated stepping probabilities from the same 2012 data and computed the scaling predicted by our theory. By the structure of the random walk model, the probabilities should scale as *P*_*r*_∝exp(*br*^1/3^). Figure 2d, which shows the empirical path probabilities (on a logarithmic scale) versus their ranks (on a cube root scale), confirms that this scaling is observed. The predicted slope of *b*_thry_=−0.8610 on that plot is close to, though mildly steeper than, the empirical slope of *b*_emp_=−0.7343. Thus, we conclude that outs sequences in American Baseball are not power law distributed, but rather follow a stretched exponential distribution, and that a simple random walk model, in conjunction with our scaling theory, is sufficient to explain their observed distribution.

As a second example, we present a symbolic dynamics analysis of the Lorenz attractor. Originally devised to model atmospheric convection, the Lorenz dynamics are given by a system of three differential equations^23^:


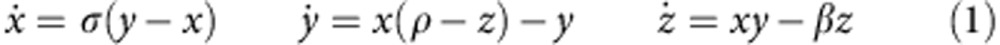


where *x*, *y* and *z* are the variables, and *σ*, *ρ* and *β* are parameters controlling the dynamics. Lorenz’s observations of the complexity of its dynamics, which are neither periodic nor stable, lead to the birth of chaos theory^24^. Figure 2e shows a trajectory of the system projected onto the *xy* plane, where two main loops in the trajectory are easily seen: a loop in the positive-*x* or right halfspace (*R*) and a loop in the negative-*x* or left halfspace (*L*). The field of symbolic dynamics^25^ includes widely used techniques for the qualitative description of continuous dynamical systems, including enumeration of possible paths and quantification of system complexity or entropy. As an example of a symbolic dynamics analysis, we numerically simulated a single very long trajectory of the Lorenz system, such that there were a total of 10^7^ halfspace loops (either *R* or *L*). We then divided that trajectory into segments based on every time it passed through the region *x*=0, *y*<0, where the Lorenz system is just entering the right halfspace *R*. Each segment thus involves one or more loops around the right halfspace, a transition from the right to left halfspaces (through the region *x*=0, *y*>0), and then one or more loops around the left halfspace, before returning to *x*=0, *y*>0. Thus, a symbolic sequence describing a segment can be written as *R*^*m*^*L*^*n*^ where *m*, *n*≥1 indicate the number of right halfspace and left halfspace loops. We then analysed the empirical frequencies of different sequences. As shown in Fig. 2f, the empirical frequencies are not consistent with power law scaling. As in the baseball example, we asked whether a simple random walk model could explain the observed scaling. We posed the model shown in Fig. 2g, estimating the transition probabilities from our long simulated trajectory. Our theory predicts that the probabilities should scale as *P*_*r*_∝exp(*br*^1/2^), which is borne out by the plot in Fig. 2h. Moreover, the empirical slope of *b*_emp_=−0.3106 is quite close to our theoretical slope of *b*_thry_=−0.3443. Thus, we conclude that a simple random walk model can explain the observed distribution of qualitative dynamical sequences of the Lorenz system.

### Quantitative analysis of power law scaling

**Figure 3:**
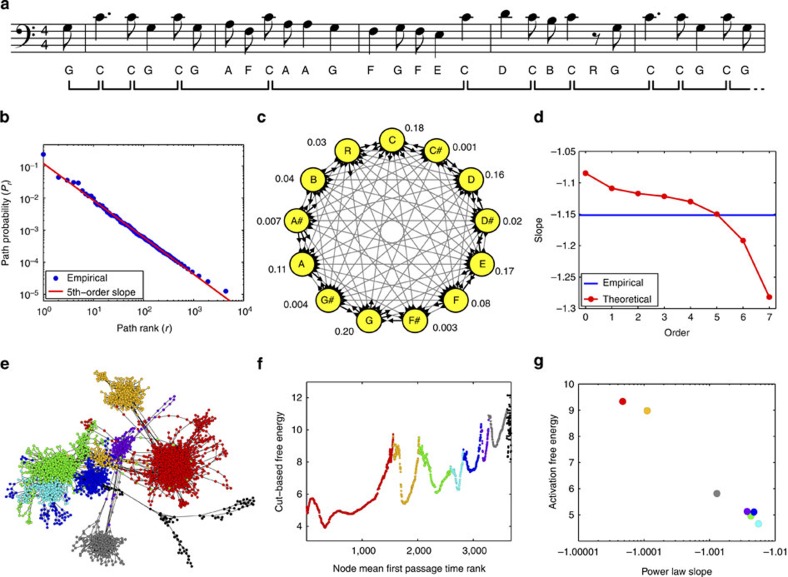
Quantitative analyses of power law scaling in folk songs and in protein-folding dynamics. (**a**) First line of the song ‘Gedenk Mit Hochgefuehl An Jene’ with accession code ‘elsass15’. Names of notes are underneath, along with the division into segments based on every occurrence on the note C. (**b**) Empirical frequencies of different segments showing clear evidence of power law scaling along with the theoretical slope predicted by a fifth-order random walk model. (**c**) Diagram of a first order random walk model built based on all 8,466 songs. Grey lines indicate possible transitions, while the lengths of outgoing arrows are proportional to transition probabilities. The notes and rest are also labelled with their overall frequencies in the data. (**d**) Predicted slope of the scaling relationship according to random walk models of different orders. An order-five model provides the best match to the empirical slope of the relationship, as obtained by linear regression. (**e**) Diagram of a 3,683-node random walk model of G-protein folding^31^. Nodes represent protein conformations and links are possible transitions, with stepping probabilities estimated by molecular dynamics simulations. Colours indicate different basins of the energy landscape (see next panel). (**f**) Nodes are ranked by their mean first passage time to the native state, and the cut-based free energy is calculated. These were manually separated into seven different energy basins (red, orange,..., grey) between which there is a sharp increase in free energy. (**g**) Although all seven power law distributions have slopes close to −1, they are not all the same. Intriguingly, the slopes appear strongly related to the activation free energies of each basin.

Our theory also leads to a more detailed and quantitative understanding in cases of power law scaling. As mentioned above, the most widely known previous result is that constructing paths by, at each step, choosing uniformly randomly from *N* nodes produces a power law path distribution with slope *b*_unif_=−log(*N*)/log(*N*−1) (ref. 16). However, are real-world examples of power law scaling quantitatively consistent with a uniform random walk model? To test this, we turned to the field of music. Like natural language, where the uniform *N*-node model originated, music contains considerable long-range correlations^26^,^27^,^28^ and complex structures^29^. Moreover, several recent analyses have uncovered various forms of power law scaling in large music corpora^27^,^28^. We downloaded the ‘Essen’ collection of 8,473 folk songs, primarily of European and Chinese origins, from the Humdrum online musical archive ( http://kern.humdrum.org). After omitting seven songs with unclear notations, we transposed the remaining 8,466 songs into the key of C. Notes such as C# and D♭ were considered as one, so that we had 13 distinct symbols: A, A#/B♭, B, C, C#/D♭, D, D#/E♭, E, F, F#/G♭, G, G#/A♭ and R (for rest). We divided each song into segments based on every occurrence of the note C, resulting in 83,436 song segments departing from and returning to the natural (tonic) tone (Fig. 3a). The empirical probabilities of the 19,449 distinct musical segments are shown plotted against their ranks in Fig. 3b, which clearly indicates power law scaling. However, the slope of the relationship is not at all consistent with a uniform-probability model. With *N*=13 distinct symbols, the predicted slope would be *b*_unif_=−1.0322, whereas the empirical best-fit line has a much steeper slope of *b*_emp_=−1.1515.

To determine whether the empirical scaling is consistent with that of a random walk, we first constructed the random walk model shown in Fig. 3c. It predicted power law scaling, but with a still-too-shallow slope of −1.1088. Reasoning that the longer-range correlations in note sequences might be a factor, we built a set of random walk models of different orders *K*=0 to 7. In an order-*K* model, the probability of the next note/rest depends on the previous *K* notes/rests in the segment. All models predicted a power law path distribution, but the predicted slopes ranged from −1.0848 to −1.2817 (Fig. 3d). Intriguingly, the predicted slopes are decreasing in the model order *K*, with the fifth-order model showing the highest consistency with the empirical slope. This finding is broadly consistent with maximum-likelihood cross-validation analysis, which favours a model of at least order 3, and equal-symbol autocorrelation analysis^30^, which favours a model of at most order 7 or 8 (see Supplementary Note 4). Thus, we conclude that the empirical scaling of these musical segments is power law and is well explained by a random walk model, but that the walk requires an approximately five-step history dependence.

As a second example, we looked at a stochastic model of G-protein folding^31^. Proper folding of proteins^2^ is crucial to their biological functions, and indeed, some proteins carry out their functions by altering their conformations under different circumstances. Conversely, a number of serious diseases involve protein misfolding, including cystic fibrosis, Alzheimer’s disease and Parkinson’s disease^32^. Protein folding can be conceptualized as a random walk on a network of possible conformations, with the relative energies of different conformations determining the probability of transitioning between them^1^,^2^,^3^,^33^,^34^,^35^. Using fine-grained molecular dynamics simulations, Scalco and Caflisch^31^ constructed a G-protein-folding model comprising 3,683 states and 27,742 possible transitions (Fig. 3e).

Transitions between basins or ‘attractors’ of the energy landscape signify important qualitative changes in the protein conformation. We used the cut-based free-energy approach to identify energy basins of the network (Fig. 3f, and colours in panel e)^31^,^36^. Then, to study the paths by which such transitions occur, for each basin we found the node with highest steady-state probability, and we calculated the 10,000 most probable paths that leave the basin (entering any other basin). All path distributions appeared power law (see Supplementary Note 5), as was also predicted by our theory based on the connectivity of transitions within basins. The predicted slopes are all close to −1, varying from −1.000047 to −1.0056. As small as that variation my seem, we wondered whether the differences might correlate to other features of the network or transition probabilities. As stated above, there is no necessary connection between the scaling slope and first passage times, mixing times and so on. However, we tried computing the free energies of activation of each basin—the negative logarithm of the steady-state flux across the basin boundary divided by the steady-state probability of the basin. Plotting those activation energies against the power law slopes for each basin, we found a nearly monotone relationship—indeed, a nearly linear relationship when the slope minus one is plotted on a logarithmic scale. To our knowledge, this is the first time that a connection has been made between the slope of a power law scaling relationship—well known from linguistics, physics, biology and so on—and activation energies—a central concept in chemical theory.

## Discussion

We have presented a new theory of the scaling of path probabilities generated by random walks on networks. Our theory implies that the distribution of path probabilities is either finite, stretched exponential or power law, depending on the connectivity of the network. This result closes a long-open question in the scaling behaviour of random sequences of symbols^14^,^15^,^16^, finally clarifying and characterizing the full set of possibilities. Moreover, our theory allows computation of the parameters of the distribution, as we demonstrated in examples drawn from sports, nonlinear dynamics, stochastic chemical kinetics and the analysis of music.

Our analyses of baseball and of the Lorenz attractor are but two examples of what we expect to be a widespread, if often overlooked, phenomenon of stretched exponential scaling. In the realm of games, there are many quantities (outs, fouls, scores and so on) that either remain the same or increase on each play; thus, we would expect their observed sequences to obey stretched exponential scaling. Similar systems also abound in epidemiology, such as the susceptible-infected-removed model of the stages of infection^37^ and many other progressive disease models; in manufacturing and logistics, where products are created or transported in a series of stages^38^; in many kinds of dissipative systems, where ‘items’ such as molecules or people survive for a limited period^39^; and so on. Thus, we expect that many instances of stretched exponential scaling can be found and will be explicable based on random walk models.

In our analysis of musical sequences, we showed that the match between empirical and theoretical scaling can be used to determine the complexity of the model, in terms of the degree of history dependence in the random walk model. In the analysis of G-protein folding, we uncovered an unsuspected connection between the exponent of power law scaling in escape paths from energy basins and the activation free energy. Because so many systems are well described by random walks on networks, from the actions of molecules^1^,^2^,^4^,^40^,^41^ to human behaviour^5^,^6^,^7^,^8^, our theory has broad potential to explain scaling phenomena. Our theory could also be used predictively to anticipate the type and possibly parameters of the path distribution based on a random walk model—potentially, even before sufficient data has accumulated to empirically observe how path probabilities scale.

## Methods

### Calculation of path distribution type and scaling parameters

To carry out the analyses in this paper, we developed a general MATLAB code that takes as input a specification of a random walk on a network. That specification includes the number of nodes in the network, identification of START and END nodes and the stepping probabilities between nodes. Our code, which is available at http://www.perkinslab.ca/Software.html, computes both the form of the path distribution and its parameters. Pseudocode for the algorithms embodied by the code are available in Supplementary Note 2.

### American baseball

We downloaded all data files describing 2012-season American Major League Baseball games from the website http://www.retrosheet.org, as a bulk zip file in late November/early December 2013. At that time, it was the most recent complete season for which data was available. We used the ‘bevent’ programme, also available at that website, to parse the data files and to output the outs sequences for each inning. To count the number of times each distinct sequence of outs occurred, we converted each outs sequence into a string, and then used the ‘unique’ function of MATLAB to count occurrences. The stepping probabilities of the Markov model in Fig. 2c are the maximum-likelihood estimates. That is, to estimate the stepping probability from *i* outs to *j* outs, we simply counted the total number *n* of at-bats that started at *i* outs and the total number of times *m* that the next at-bat started at *j* outs. The empirical ratio *m*/*n* is the maximum-likelihood stepping probability estimate.

### Symbolic dynamics of the Lorenz system

In analysing the Lorenz dynamics, we employed the parameters *σ*=10, *ρ*=28 and *β*=8/3, which are the standard choices for which the dynamics are known to be chaotic. We simulated one very long trajectory from the inital condition *x*=*y*=*z*=1, using the ‘ode45’ function of MATLAB, with default parameters. That trajectory was long enough to yield 10^7^ total qualitative states (left *L* or right *R* halfspace). These were then divided into segments at every *L*→*R* transition, constituting the paths from START to END in our symbolic dynamics analysis. The long trajectory provided 2,145,793 paths, ranging in length from just two steps (*RL*) to 35 steps (*R*^18^*L*^17^). As with the baseball example, we estimated the stepping probabilities of our random walk model simply by counting the empirical frequencies of different transitions in the data.

### Essen folk songs collection

We downloaded the ‘Essen’ folk song collection in the form of ‘kern’ files. The kern format gives a key signature for each song, as well as the sequence of notes (pitch and duration) or rests. Seven songs had unclear notations in their kern files and were omitted from the analysis: china01, china07, deut1328, han0089, han0351, han0404 and han0953. After transposition to the key of C, we did not discriminate between notes in different octaves. For instance, middle C, high C and indeed any other C, were all coded just as C. As stated above, the paths for our analysis were obtained by dividing the song based on every occurrence of the note C.

To estimate a *K*-order random walk model for a specific value of *K*, we first identified all the unique *K* tuples occurring in paths, along with the note sequences in paths less than *K* notes long. So, for instance, suppose *K*=5. A song segment such as CEGECEGC contains the *K* tuples CEGEG, EGECE, GECEG and ECEGC. A shorter segment like CEC would be considered to generate the single *K*-tuple CEC, even though this is really shorter the *K* notes long. The nodes of the network correspond to all the unique *K* tuples thus identified, along with special START and END nodes. The stepping probabilities among these nodes are then computed to be proportional to the empirical frequency of observed transitions. For instance, the segment CEGECEGC contains the transitions START→CEGEC, CEGEC→EGECE, EGECE→GECEG, GECEG→ECEGC and ECEGC→END. The short segment CEC contains the transitions START→CEC and CEC→END. Such short segments cannot participate in cycles, and thus do not end up affecting the asymptotic scaling. Nevertheless, we included them in our model for completeness.

### G-protein-folding model

Scalco and Caflisch^31^ provided us their G-protein random walk model based on molecular dynamics computations described in their paper. All links in the model are bidirectional because protein conformational changes are reversible. However, the probability of stepping from node *i* to *j* is not generally the same as the probability of stepping from node *j* to *i*. The network comprises a single, strongly connected component and the random walk posesses a unique well-defined steady-state distribution. Following the lead of Scalco and Caflisch, we computed the steady-state distribution for the walk and designated the single most probable node under that distribution as the ‘native’ or folded state.

The cut-based free-energy approach for identifying approximate ‘energy basins’ of the network^31^,^36^ works as follows. First, we compute the mean first passage time from each node *i*≠1 to node 1 (the native state)—that is, the expected time it takes for the random walk, if it starts at node *i*, to reach the native state. This can be computed by a relatively efficient and simple dynamic programme. Next, we sort the nodes by increasing mean first passage time. Intuitively, nodes with higher mean first passage time are ‘farther’ from the native state, at least in terms of the random walk. Closely connected nodes are expected to have similar mean first passage times. Then, for each node *i*≠1, we imagine dividing, or cutting, the network into two parts: on one side are the nodes with first passage time smaller than *i*’s, on the other side are nodes with first passage time greater than or equal to *i*’s. We compute the steady-state flux across this cut. (The steady-state flux across an arc *i*→*j* is the steady-state probability of *i* times the transition probability from *i* to *j*. The steady-state flux across the cut is the sum of the steady-state fluxes of all arcs from one side to the other.) Then, for all nodes *i*≠1, we plot the negative logarithm of the steady-state flux, which is also called the cut-based free energy, against the rank of node *i* in order of increasing mean first passage time. We visually inspect that plot to separate the nodes into energy basins, by looking for local free-energy maxima separating broad regions of lower free energy. Carrying out this procedure for the G-protein model, we were able to divide the network into seven major energy basins, with a relatively small number of extra nodes that did not clearly comprise a basin. Although the ‘extra’ nodes are closely connected in the network, the cut-based free-energy analysis did not indicate a cohesive basin.

For each basin, we then analysed the exit dynamics in the following way. First, we created a new random walk by selecting as nodes only those within the basin, plus an additional END node. A step from from node *i* to node *j* within the basin was assigned the same probability as in the original walk. If, in the original walk, node *i* allowed steps outside of the basin, then we added an arc from *i* to END with stepping probability equal to the sum of those original outside steps. The node in the basin with highest steady-state probability (under the original walk) was designated as the START node. We then analysed paths from START to END as in all other examples.

## Author contributions

T.J.P., E.F., R.E. and L.G. conceived the study and contributed to writing the manuscript. T.J.P. wrote the analysis software and conducted the computational analyses.

## Additional information

**How to cite this article:** Perkins, T. J. *et al.* A scaling law for random walks on networks. *Nat. Commun.* 5:5121 doi: 10.1038/ncomms6121 (2014).

## Supplementary Material

Supplementary Information Supplementary Figures 1-5, Supplementary Notes 1-5, Supplementary References

## Figures and Tables

**Figure 1:**
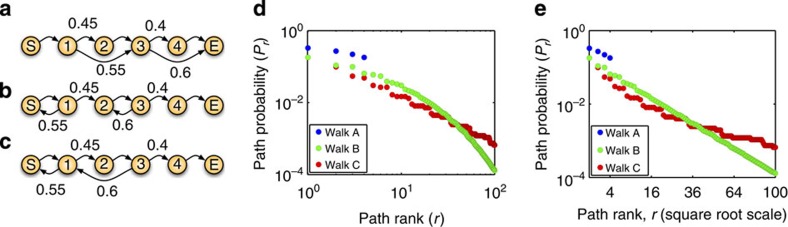
The path distribution for a random walk on a network may be finite, stretched exponential or power law. (**a**–**c**) Graphical depiction of three different random walks on networks, all having the same set of nodes and transitions probabilities, but with some arcs having different endpoints. Arcs without numbers are probablity-one transitions. (**d**) A log–log plot of the probabilities of different paths from S to E, under the walks shown in **a**–**c**, where *P*_*r*_ denotes the probability of the *r*^*th*^ most probable path from S to E. Walk A allows only four possible paths from S to E, so its distribution is finite. For walk C, the approximate linearity of *P*_*r*_ with *r* on the log–log plot suggests that the path distribution is power law. The curvature of the points for walk B is inconsistent with a power law path probability distribution. (**e**) When log probabilities are plotted against the square root of rank, the points for walk B are approximately collinear, indicating a stretched exponential path probability distribution.
