# Supplementary material for: A scaling law for random walks on networks
Source: Nat Commun. 2014 Oct 14;5:5121. doi: 10.1038/ncomms6121 (PMC4214407; doi:10.1038/ncomms6121)
Supplement: Supplementary Information — Supplementary Figures 1-5, Supplementary Notes 1-5, Supplementary References [file ncomms6121-s1.pdf]

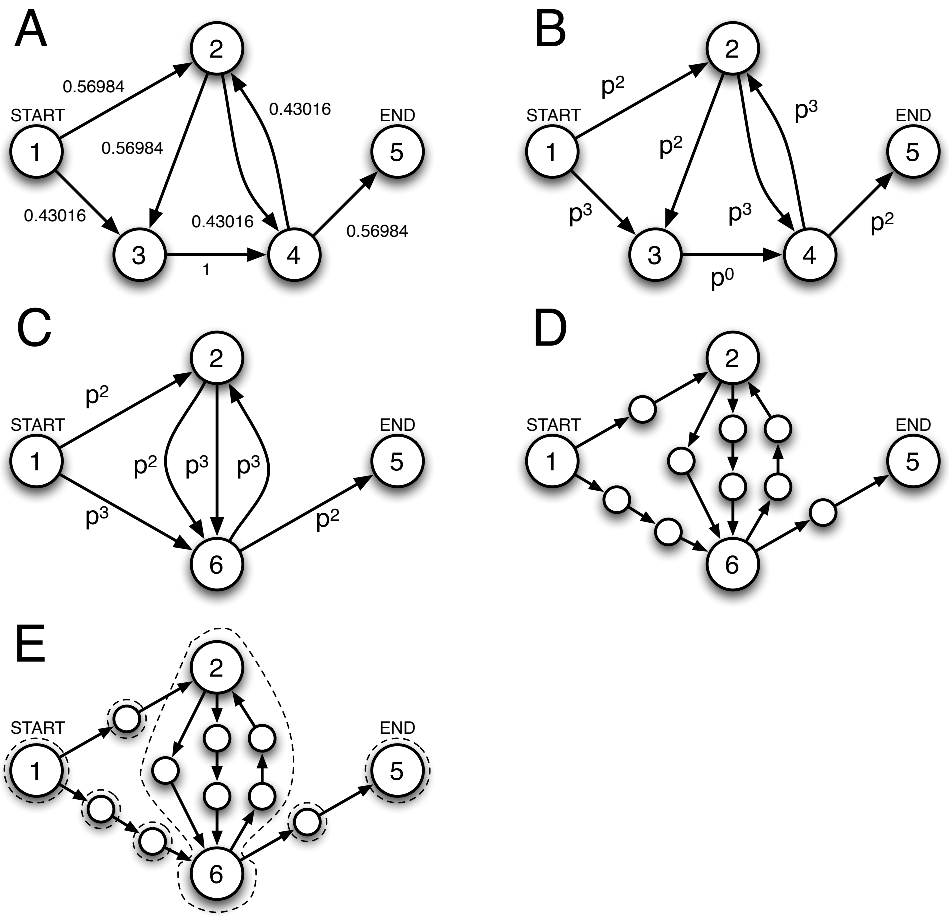

Supplementary Figure 1: An example walk and demonstration of the steps in deriving the scaling law. (A) Example network, with arcs labelled by stepping probabilities. The walk starts in node 1 and ends in node 5. (B) The same network, but with transition probabilities re-expressed as integer powers of a base probability $p \approx 0.7548776$, which solves $p^2 + p^3 = 1$. Our scaling law does not require transition probabilities to be exact integer powers of some base probability p, however, it makes the arguments we present here more straightforward. See [1] for details on how to handle the more general case. (C) Any transition with probability 1, say from node i to node j, is eliminated by collapsing the two into a new node k. All incoming arcs to the old nodes i and j are redirected to node k, while all outgoing arcs from the old node j are copied over to node k. In this network, nodes 3 and 4 are collapsed into a new node 6. After this transformation, each arc has probability p^n for some $n > 0$. (D) Each arc with probability p^n is replaced by a series of n arcs. The crucial benefit of this transformation is that every path with probability p^n in the original network has a corresponding path of length n in the new network D. Counting paths of a certain probability in network A thus corresponds to counting paths of a certain length in network D—a problem that is well understood. (E) Strongly connected components of the network, indicated by dashed lines, are identified to determine how the number of paths grows with path length. In this case, one component supports exponential growth, so that the overall distribution of path probabilities will be powerlaw. The slope of the powerlaw depends on the base probability p and the exact rate of exponential growth of the number of paths.

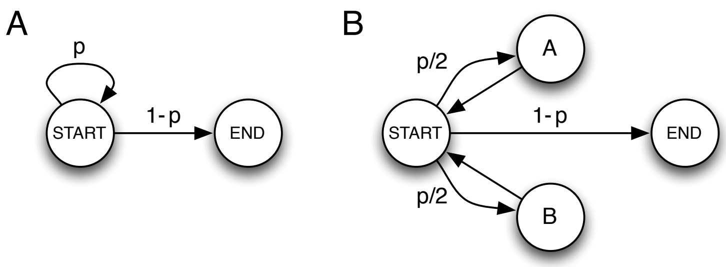

Supplementary Figure 2: Parameterized random walks demonstrating that nearly arbitrary scaling slopes can be achieved. (A) Mono-cyclic case. (B) Multi-cyclic case.

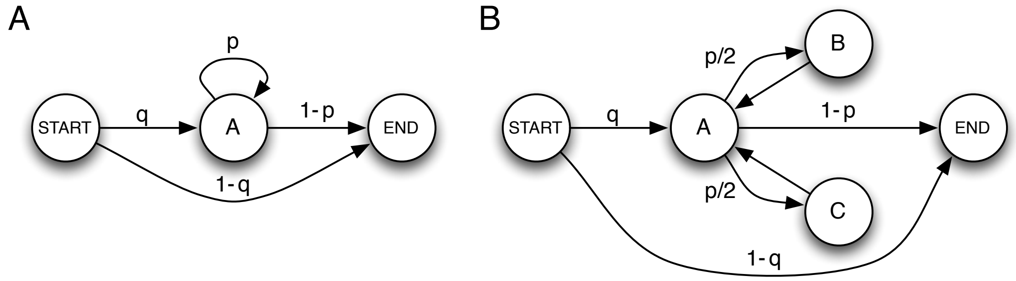

Supplementary Figure 3: Parameterized random walks demonstrating that expected first-passage time and scaling slope need not be related. (A) Mono-cyclic case. (B) Multi-cyclic case.

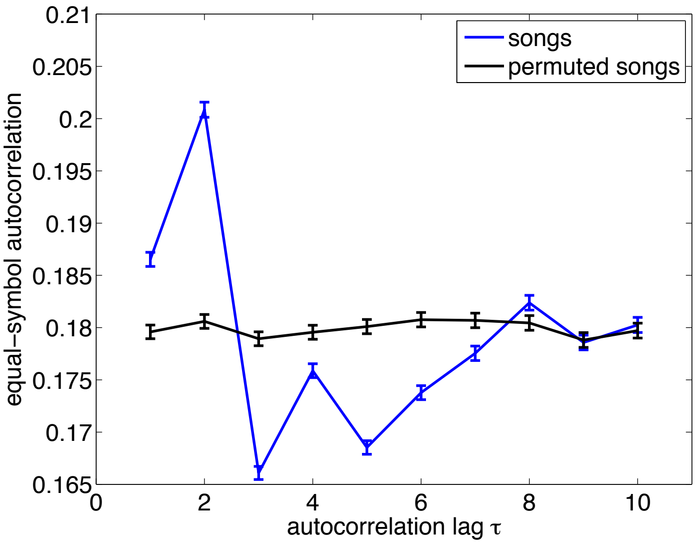

Supplementary Figure 4: Equal-symbol autocorrelation analysis of the songs in the Essen collection suggest history dependence of no more than 7 steps.

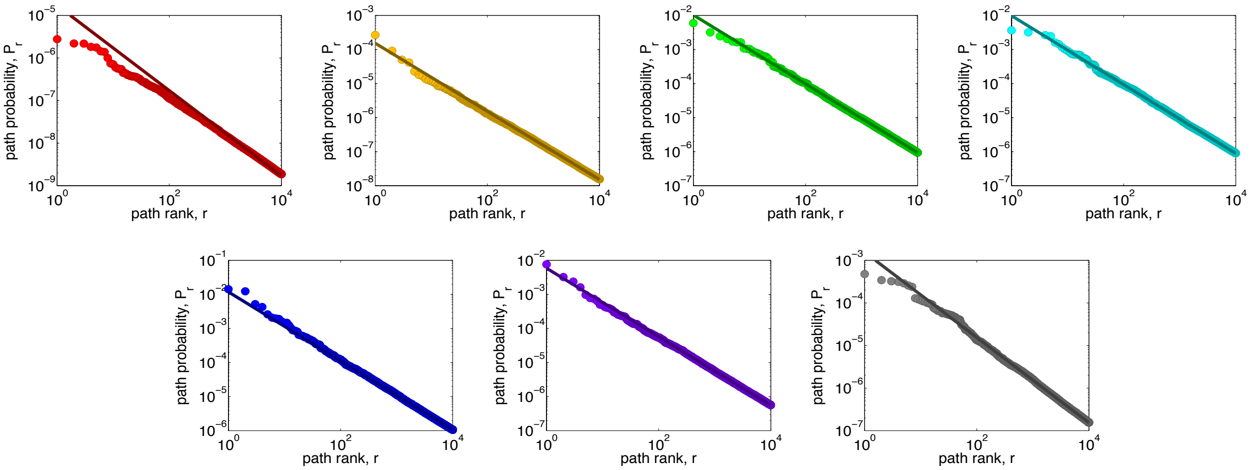

Supplementary Figure 5: For each of the seven energy basins of the G-protein model, plots of the log probability versus log rank for escape paths indicate powerlaw scaling. Solid lines are predicted slopes, based on our scaling theory.

Supplementary Note 1: Justification for the scaling law

Our scaling law generalizes previous work by Mandelbrot [2], Miller [3] and Li [4]. The latter two works assumed all transitions are equiprobable, and that any node of the network could come next in the random walk. The former work sketched arguments that allowed for non-equiprobable transitions as well as restrictions on which nodes could be stepped to from each node. However, it focussed on a subset of the powerlaw cases. No previous work has addressed all possible random walks on networks.

Here, we sketch the main argument establishing our new scaling law—in particular, the argument for the three possible types of path distributions and the circumstances in which they occur. We describe how, given a walk on a network, we can successively transform the problem into a form that allows us to establish our law. We also demonstrate the steps of the transformation on an example walk. Rigorous proof of our scaling law is quite technical, so we must refer the interested reader to our recent paper for details [1]. Here, we present arguments in more palatable form. We also alleviate a relatively minor technical obstacle, but one necessary for a law that covers all possible random walks, by providing a mechanism to handle probability-one transitions in the network.

Suppose we are interested in a walk on a network with nodes $1 \dots N$. One of these nodes is designated as the START of the walk, and one is designated as the END of the walk. Because the walk ends upon reaching the END node, we assume that the END node has no outgoing arcs. Let P_{ij} be the probability that the walk steps from node i to node j. Supplementary Figure 1A gives an example walk that we will use to demonstrate the steps of our derivation.

Most previous analyses of path distributions have assumed all possible steps happen with equal probability. We do not make any such restriction. As a matter of convenience, however, we will assume that we can represent all the non-zero transition probabilities as $P_{ij} = p^{k_{ij}}$ for some non-negative integers k_{ij}. That is, all non-zero transition probabilities are integer powers of some "base" probability p. See Supplementary Figure 1B for an example. This assumption makes the arguments presented here more direct, but is not required by our theory; the scaling law holds for arbitrary probabilities [1].

Now, suppose there is a path P from START to END of probability p^n. What is the rank, r, of path P? Its rank is simply one more than the number of paths from START to END of strictly greater probability. If there are M such paths, then $r = M + 1$, because path P is (at least tied for) rank $M + 1$. Thus, our main task will be to count, for a given probability level p^n, how many paths there are of probability greater than p^n.

Step1: Elimination of probability-one transitions. The first step in our analysis is to eliminate, or "collapse", any probability-one transitions. The reason for this will become clear shortly. For every pair of nodes (i, j) where $P_{ij} = 1$, the nodes i and j should be coalesced into a single node. To be specific, we delete nodes i and j from the network, and we create a new node k. The incoming arcs to node k include all the incoming arcs to node i and all the incoming arcs to node j except the arc from node i. The outgoing arcs from node k are precisely the same as the outgoing arcs from node j. In this procedure, if node i is the START node, then the new node k should be re-designated as the START node. Likewise, if node j is the END node, then the new node k is re-designated as the END node. When the network contains multiple pairs (i, j) for which $P_{ij} = 1$, then the node pairs may be coalesced in any order. Each coalescing operation reduces the number of probability-one transitions, so after at most N such operations, all probability-one transitions will have been removed from the network.

Supplementary Figure 1C gives an example of the elimination of probability-one transitions. Observe that it is possible, as we have included in the example, for the coalescing of two nodes to result in a node l having two separate arcs to the new node k. In this case, node 2 originally has links to nodes 3 and 4. However, nodes 3 and 4 are coalesced, after which node 2 has two distinct links to the new node 6, each of which is followed with a different probability. This is allowed, and henceforth paths through the network must be thought of as sequences of arcs rather than as sequences of nodes.

A crucial feature of our elimination of probability-one transitions is that it changes neither the number nor probabilities of different paths from the START node to the END node. We omit careful proof of this fact, although we hope it is self-evident. In essence, any path which previously traversed the $i \to j$ link as follows: START $\to \cdots \to x \to i \to j \to y \to \cdots \to$ END, instead now follows the path: START $\to \cdots \to x \to k \to y \to \cdots \to$ END.

Step 2: Expand single transitions into chains. In the second step of our analysis, we produce a new network in which every probability p^n arc in the network is replaced by a series of n arcs through intermediate or "dummy" nodes. For instance, in Supplementary Figure 1D, we see that the probability p^2 arcs in the network of panel C are replaced by a pair of arcs which traverse a single new node (shown as a smaller circle) that has been added to the network. The probability p^3 arcs are replaced by a series of 3 arcs traversing two new nodes. Because Step 1 of our analysis ensures that each arc has probability p^n for some strictly positive integer n, we are assured that the chains of arcs in Step 2 have at least length one. This transformation does not change the number of paths between any of the non-dummy nodes (1, 2, 5 and 6, in the example of Supplementary Figure 1). However, it changes the lengths of those paths, and it does so in a way that is particularly helpful for our analysis. In particular, every path of probability p^n in our original network now has a corresponding path of length n in our transformed network. Conversely, every path of length n between non-dummy nodes in the new network corresponds to precisely one probability p^n path in the original network. Because of this, counting paths of a given probability in our original network is equivalent to counting paths of a given length in our expanded network. There is a large, established literature on counting paths in networks by length, and these tools help us, at least in part, to establish the form and parameters of the path probability distribution.

Step 3: Reachability analysis to identify "live" nodes. The next step of the analysis is to conduct a reachability analysis of the network. We say node i can reach node j if there is a path comprising at least one arc that leads from i to j. This definition includes the special case where $i = j$; thus, we only say i is reachable from itself if there is a path which leaves and returns to node i. Reachability can be established by several efficient algorithms, as is well known [5]. With the exceptions of the START and END nodes themselves, we discard any nodes that are not reachable from START, or from which the END is not reachable. Such nodes cannot play any part in a path from START to END, and hence are irrelevant to the path distribution. The network in Supplementary Figure 1D has no such nodes, but in general, this is possible. The nodes that remain, which participate in paths from START to END, we call "live" nodes.

Step 4: Analyze strongly connected components to determine overall type of path distribution. After restricting attention to live nodes, we identify the strongly connected components of the network. A strongly connected component is either: (1) a single node, which is not reachable from itself, or (2) a set of nodes, each of which is reachable from every other node in the set. Option 2 includes the special case of a single node with a self-loop. Supplementary Figure 1E shows the strongly connected components of our example walk. (For the sake of brevity, we will sometimes use just "component" to stand for strongly connected component.) It turns out that

the components of type 2, have two important subtypes. In one case, the set of one or more nodes, call them $x_1, x_2, \ldots, x_m$, are connected in a simple cycle, without any other links between them. That is, there is a single arc from each x_i to x_{i+1}, and a single arc from x_m to x_1. Thus, the only path from a node x_i back to itself is the path that loops through every node in the component. On the other hand, suppose that there is at least one additional arc between two nodes in the component. This includes the special case of multiple arcs between two nodes, which can result from the elimination of probability-one transitions. In this case, there are at least two distinct paths that may return to node x_i. In this case, we will say that the component is "more than a cycle".

The overall rate at which the number of paths from START to END grows depends on the types of strongly connected components in the network.

Case 1. The network is *acyclic* if all components are of type (1) above—single nodes not reachable from themselves. In this case, no path from START to END can contain a loop. Thus, there are only finitely many possible paths possible from START to END. In this case, the path distribution has no asymptotic behaviour to characterize.

Case 2. The network is *mono-cyclic* if it has at least one component of type (2) above—one or more nodes that are reachable from each other—but all such components are simple cycles. In this case, the number of paths from START to END grows polynomially in path length L. To see this, let us denote the type (2) components as C_1, C_2, ..., C_m. Let the sizes of those components be $l_1, l_2, \ldots, l_m$ with $l_{\max} = \max(l_1, l_2, \ldots, l_m)$. Some components may be reachable from others, but if C_j is reachable from C_i, then C_i cannot be reachable from C_j. If it were, then the two would actually be part of a single strongly connected component. Let us suppose, for convenience, that the components are listed in topological-sorted order. By this, we mean that if C_j is reachable from C_i, then $j > i$. As such, there are at most 2^m possible sequences of components that may be visited on any path from START to END—each of the C_i either is or is not visited as part of a path. Depending on the structure of the network, many of these may in fact be impossible. In any case, let us consider a particular sequence of n distinct components that may be visited on a path from START to END. As a function of maximum path length L, how many paths are there that visit those n components in that order? We claim the answer is $\Theta(L^n)$. Formally, this means that there exists an integer $L^* > 0$ and there exist real constants $a, b > 0$ such that for all $L \geq L^*$, the true number of paths is at least aL^n and at most bL^n.

First, we argue that the number of possible paths of length $\leq L$ that visit a specific sequence of n components is at most of the order L^n. Any such path can be divided into the steps that lead it to the first component, the steps within the first component, the steps that lead it to the second component, the steps within the second component, etc. Let us assume there are $p_{0,1}$ possible paths from START to the first component, $p_{1,2}$ possible paths from the first component to the second, ..., $p_{n,n+1}$ possible paths from the last component to the END. These numbers depend on the structure of the network, but are independent of the maximum path length L. The total numbers of ways of transiting to and from the components is $p^* = \prod_{i=0}^{n} p_{i,i+1}$. Now, the START node may actually be within the first component. But all remaining transitions between components or to the END node require at least one step. This leaves no more than $L - n$ steps that may occur strictly within the components. A basic result from combinatorics is that there are $\binom{a+b-1}{b-1}$ ways to distribute a indistinguishable objects into b distinct containers [6]. In the present case, we can consider the $L-n$ remaining steps as objects. There are $n+1$ containers into which to put them—n components, plus an extra container for steps that go "unused". (Remember, L is the maximum path length; for shorter paths, the extra steps go into the "unused" container.) As such, there are

at most $\binom{(L-n)+(n+1)-1}{(n-1)+1} = \binom{L}{n} \leq L^n/n!$ ways of distributing those steps among the components or leaving them unused. In total then, there are no more than $L^n(p^*/n!)$ possible paths from START to END that traverse a particular sequence of n components.

Next, we argue that the number of possible paths of length $\leq L$ through n specific components is at least of the order L^n. Again, imagine these paths divided into segments between components and segments within components. There are at most $n+1$ segments outside of components: one from START to the first component, $n-1$ between components, and one from the last component to END. Each of these segments can be carried out by at least one path, and can take no more than N steps, where N is the total number of nodes in the network. Therefore, at least $L - N(n+1)$ steps are available to take place within strongly connected components. Now, the entry and exit nodes to the components may be constrained. But, to get from any entry to any exit point takes at most $l_{\max}$ steps. That still leaves at least $L - N(n+1) - nl_{\max}$ steps in the path. To go around the cycle of nodes that comprises one component takes at most $l_{\max}$ steps. Therefore, the remaining steps allow for at least $L' = \lfloor (L - N(n+1) - nl_{\max})/l_{\max} \rfloor$ loops around any component. These loops can be distributed arbitrarily among the n components or go unused (resulting in a shorter path) in at least $\binom{L'+n}{n} \geq (L')^n/n! \geq bL^n$ ways, for some constant b and for sufficiently large L.

Summarizing the previous two paragraphs, then, the number of paths of length less than or equal to L that go through a specific sequence of n components is bounded above and below by something proportional to L^n. Therefore, the number of paths is $\Theta(L^n)$. The total number of paths from START to END of length $\leq L$ is obtained by adding together the paths from all possible sequences of components that may be visited on any path from START to END. If k is the maximum number of cycle-components that may be encountered on any path from START to END, the total number of paths therefore grows as $\Theta(L^k)$.

In terms of the path distribution, consider a path from START to END of length L. Recall that such a path has probability p^L. The rank of such a path depends on how many paths there are that are shorter. By the above arguments, this number is $r \approx cL^k$ for some constant c. Therefore, the log probability of that path is $\log P_r = L \log p$ and the kth root of its rank is $r^{1/k} \approx c^{1/k}L$. The ratio $\log P_r / r^{1/k}$ is approximately constant, and the path probability as a function of rank, P_r, is approximately a stretched exponential. This establishes the mono-cyclic case of our scaling law.

Case 3. The network is *multi-cyclic* if there is at least one strongly connected component that is more than a simple cycle. If there is exactly one such component, then the number of paths from START to END of length less than or equal to L grows exponentially. Why is this? Getting from START to the component that is more-than-a-cycle takes at most N steps, and at most N steps are needed to get from there to END. That leaves at least $L - 2N$ steps that may be used within the component. Frobenius-Perron theory [7] tells us that the number of paths within the component grows at $\lambda^{L-2N} = \Theta(\lambda^L)$, where λ is the largest eigenvalue of the adjacency matrix of the component. (Note that "adjacency matrix" here means a matrix that *counts* the number of arcs from each node i to each node j, so its entries are nonnegative integers.)

Now, suppose there are multiple such components in the graph, and suppose in particular that it is possible to visit n of them along some path from START to END. Let $\lambda_1, \ldots, \lambda_n$ be the largest eigenvalues of the adjacency matrices of those components, and let $\lambda_{\max} = \max(\lambda_1, \ldots, \lambda_n)$. How many paths are there from START to END of length $\leq L$? Again, there are transitions between the components, but the numbers and lengths of these do not depend on L. The key question is how the remaining steps (somewhere between $L - Nn$ and $L - n$) are distributed among the components. Clearly, we can generate $\Theta(\lambda_{\max}^L)$ paths, by putting all those extra steps into the single component with largest eigenvalue. In fact, even if all components have that same eigenvalue, the best we can

do is distribute the approximately-L remaining steps among them in $\binom{L}{n}$ ways. At most, that gives us $\Theta(L^n \lambda_{\max}^L)$ paths, which is $\mathcal{O}((\lambda_{\max} + \epsilon)^L)$ for any $\epsilon > 0$. Therefore, the total number of paths from START to END of length less than or equal to L is between $\Theta(\lambda_{\max}^L)$ and $\mathcal{O}((\lambda_{\max} + \epsilon)^L)$ for any $\epsilon > 0$. Given this, a length L path has log rank $\log r \approx L \log \lambda_{\max}$ and log probability $\log P_r = \log p^L = L \log p$. As a result, the ratio $\log P_r / \log r$ is approximately constant for large L, or equivalently, we have an approximate powerlaw relationship between P_r and r. This establishes the multi-cyclic case of our scaling law.

Supplementary Note 2: Computing path distribution type and parameters

As stated in the main text and again in the previous note, three types of path distributions are possible: finite, stretched exponential and powerlaw. Intuitively, the categorization of a random walk and computation of its parameters could be done based on an "expanded" network, as shown in Supplementary Figure 1D. However, some walks may not correspond exactly to any expanded network—because their transition probabilities may not be integer powers of some base probability p. In other cases, those integer powers might be so large as to result in an enormous (if sparse) network, which could be computationally awkward. Fortunately, we need not actually construct the expanded network to determine the type of the distribution and its parameters. Rather, computational shortcuts allow us to establish the form of the path distribution and its parameters much more directly from the network structure and the probabilities with which different arcs are followed. We describe the necessary computations below. MATLAB code implementing the computations is available on the web, at `http://www.perkinslab.ca/Software.html`.

Determining the type of the distribution. To analyze a particular random walk, we begin by computing reachability of every node from every other node. Let Z be the set of all nodes that are reachable from the START and from which the END can be reached. If no node in Z is reachable from itself by a path of one or more steps, then we are in the acyclic case / the path distribution is finite. Otherwise, we compute the strongly connected components among the nodes in Z. If all of the components are simple cycles (i.e., each node has an arc to precisely one other node in the component), then were are in the monocyclic / stretched exponential case. Otherwise, we are in the multicyclic / powerlaw case. All of these reachability-based computations are well known in graph theory [5], so we do not further detail them here. In our MATLAB code, we use the `graphtraverse` function to assess reachability, and the `graphconncomp` function to extract the strongly connected components.

Parameters for stretched exponential path distributions. If the path distribution is determined to be of the stretched exponential type, so that $\log P_r \approx br^{1/k}$, then we desire to compute the constants b and k. To do this, let our random walk be on a network $G = (V, E)$ where $V = \{v_1, \ldots, v_m\}$ are nodes and $E \subseteq V \times V$ are directed edges. Let the stepping probabilities be P_{ij}, where $P_{ij} > 0 \iff (v_i, v_j) \in E$. For simplicity of notation, let us assume that the graph G has already been restricted to the set Z of vertices that are reachable from the START and from which the END can be reached. Nodes not in this set have no influence on the path distribution.

Now, let us construct a new network $G' = (V', E')$ with $V' = \{u_1, \ldots, u_n\}$ in the following manner. First, the END node of the random walk on G gets a vertex in V'. Second, every strongly connected component in G gets a single corresponding vertex in V'. Third, every other node in V gets its own vertex in V'. Implicitly, this three-step construction defines a surjection $f : V \to V'$. For $u_i, u_j \in V'$, let us define $N(u_i, u_j) = |\{(v_1, v_2) \in E \text{ such that } f(v_1) = u_i \text{ and } f(v_2) = u_j\}|$. In words, $N(u_i, u_j)$ is the total number of arcs in graph G from the nodes corresponding to u_i, to any of the nodes corresponding to u_j. In network G', for $u_i \neq u_j$, we include an edge $(u_i, u_j) \in E'$ if $N_{i,j} > 0$. We do not include an edge (u_i, u_i) in G' even if $N_{i,i} > 0$. Finally, for vertices u_i that correspond to a cycle in the original graph G, let $W(u_i)$ be the sum of the negative log probabilities of the steps around the cycle.

To obtain our scaling constants, we associate two values, A_0 and A_1, to each vertex in G'. The quantity A_0 is related to the transition probabilities. The quantity $A_1(u_i)$ gives the maximum number of cycles that may be encountered along any path in G from a vertex in $f^{-1}(u_i)$ to the

END. We compute both values using the dynamic program described in Algorithm 1.

```
# Initialization:
A_0(u) ← 0 for all u ∈ V'
A_0(f(END)) ← 1
A_1(u) ← 0 for all u ∈ V'

# Dynamic programming iterations:
for Iter = 1 to n do
    for u ∈ V' do
        U ← {u' ∈ V' such that (u, u') ∈ E' and A_0(u') > 0}
        if U ≠ ∅ then
            Temp1 ← max_{u'∈U} A_1(u')
            U' ← arg max_{u'∈U} A_1(u')
            if N(u, u) > 0 then
                A_1(u) ← Temp1 + 1
                A_0(u) ← (∑_{u'∈U'} N(u, u')A_0(u')) /(W(u)A_1(u))
            else
                A_1(u) ← Temp1
                A_0(u) ← ∑_{u'∈U'} N(u, u')A_0(u')
            end
        end
    end
end
```

Algorithm 1: Algorithm for computing parameters of a stretched exponential path distribution.

Once the algorithm has completed, the scaling constants can be obtained as $k = A_1(f(START))$ and $b = -A_0(f(START))^{-1/A_1(f(START))}$. In particular, b will be the slope of the line relating P_r with r, when P_r is plotted on a logarithmic axis and r is plotted on a kth-root axis. The base of the logarithm in the plot (e.g. base-10) should be the same as the base of the logarithm used to compute $W(u)$.

Parameters for powerlaw path distributions. If the path distribution is powerlaw, where $\log P_r \approx b \log r$, the parameter b can be obtained by analyzing the strongly connected components of the walks individually, and then combining the results, as described in part one of this document. Again, because an "expanded" graph, as seen in Supplementary Figure 1D, can be unwieldy, or because the transition probabilities may not be exactly integer powers of any base probability, we provide an alternate mechanism that avoids the expanded graph [1]. Also, for simplicity of discussion, we assume we have already restricted attention to nodes that are reachable from the START and from which the END is reachable.

First, let us focus on any one individual strongly connected component. Suppose this component has n nodes, and let P_{ij} for $i, j \in \{1, \ldots, n\}$ be the transition probabilities among them. Let us define a family of matrices, $M(\beta)$, indexed by real parameter β, as follows:

$$M_{ij}(\beta) = \begin{cases} 0 & \text{if } P_{ij} = 0 \\ P_{ij}^{-\beta} & \text{if } P_{ij} > 0 \end{cases}$$

For any given value of β, $M(\beta)$ has a set of eigenvalues. Let $\lambda_1(\beta)$ be the eigenvalue of $M(\beta)$ with largest real component. In our MATLAB code, we use the `eigs` function to compute the largest

eigenvalue. Then, let β^* be the value of β such that $\lambda_1(\beta)$ has real component equal to 1. To find this β^* we perform a binary search, beginning with upper and lower bounds on β^*. We repeatedly compute the midpoint β_{mid} of our upper and lower bounds, and evaluate the largest eigenvalue of $M(\beta_{mid})$. If it is larger than 1, we reset our upper bound to be β_{mid}. If it is smaller than one, we reset our lower bound to be β_{mid}. In this way, we rapidly home in on β^*, to whatever precision is desired.

Now, suppose the original walk has m strongly connected components, and we have analyzed each one to obtain $\beta_1^*, \ldots, \beta_m^*$. Let $\beta^{**} = \min_{i \in \{1,\ldots,m\}} \beta_i^*$. Then, the scaling constant b in the relationship $\log P_r \approx b \log r$ is $b = 1/\beta^{**}$.

Supplementary Note 3: On the scaling slope and other random walk statistics

There are Markov chains with (nearly) arbitrary scaling slopes. As stated in the main text, powerlaw scaling slopes are often found to be near -1. In the case of the uniform, memoryless random walk, where each step leads with equal probability to one of N nodes, one of which is the END node, the slope is $-\log(N)/\log(N-1)$ [4]. This converges to -1 rather quickly. For $N=5$, the slope is -1.16, and for $N=10$ it is -1.05. Slightly more generally, the random walk can be on a graph with M nodes, as long as at each step there are N possible next nodes from each, one of which is the END node. The total size of the graph, M, is not relevant. Of course, these are simplistic models that would not be accurate for many real-world examples. Nevertheless, one could conjecture that if, in some suitable "average" sense, a system has N possible next nodes, then it might have a similar slope, and that this might explain the frequency of powerlaw slopes near -1 in real-world examples.

Regardless, here we argue that such slopes are not in any way necessary. We describe two parameterized Markov chains, one for the mono-cyclic case and one for the multi-cyclic case, that allow us to achieve any feasible slope. Let us begin with the mono-cyclic chain, which is shown in Supplementary Figure 2A. For $K=1,2,3,\ldots$, this chain allows precisely one length-K path from START to END, and that path is the Kth most probable. It has probability $p^{K-1}(1-p)$. Thus, the slope of $\log P_r$ versus r (since this mono-cyclic chain allows at most one cycle on any path from START to END), is

$$\lim_{K\to\infty} \frac{\log(p^{K-1}(1-p))}{K} = \lim_{K\to\infty} \frac{(K-1)\log p + \log(1-p)}{K} = \log p \ . \tag{1}$$

Because p can be anything in the open interval $(0,1)$, the scaling slope can be anything in the open interval $(-\infty, 0)$.

Next, we consider the multi-cyclic/powerlaw example given in Supplementary Figure 2B. For $K=1,2,3,\ldots$, this chain has 2^{K-1} paths of length $2K-1$ from START to END. The probability of such a path is $(p/2)^{K-1}(1-p)$, and it is tied for rank $r=2^{K-1}$. Therefore, the scaling slope is

$$\lim_{K\to\infty} \frac{\log((p/2)^{K-1}(1-p))}{\log 2^{K-1}} = \lim_{K\to\infty} \frac{(K-1)(\log p - \log 2) + \log 1 - p}{(K-1)\log 2} = \frac{\log p}{\log 2} - 1 \tag{2}$$

Because p can be anything in the open interval $(0,1)$, the scaling slope can be anything in the open interval $(-\infty, -1)$.

Relationships between the path distribution and first-passage times. The first-passage time distribution of a random walk on a network describes the probability that a walk goes from START to END in exactly s steps, for any $s=0,1,2,\ldots$. For acyclic networks, the path distribution is finite and the first-passage time distribution is finite. By contrast, for mono-cyclic or multi-cyclic networks, both the path distribution and the first-passage time distribution are infinite. Thus, at this coarsest level of analysis, there is a relationship between the path distribution and the first-passage time distribution. However, for both mono- and multi-cyclic networks, the first-passage time distribution is approximately exponentially distributed [7]. Thus, the shape of the path distribution is a finer distinction, dividing networks into three categories (finite, stretched exponential, and powerlaw), whereas the first-passage time distribution divides networks into two categories (finite and exponential).

In either the mono-cyclic or multi-cyclic case, there is no necessary relationship between the expected first passage time and the slope of the path distribution. For example, consider the network walk shown in Supplementary Figure 3A. It is a modified version of the walk in Supplementary Figure 2A, and has the same scaling slope of $\log p$. The initial probability-q branch to the mono-cycle versus the probability-$(1-q)$ branch directly to END does not affect the scaling slope. It affects the expected first passage time, however. The expected first-passage time from START to END is $1 + q/(1-p)$. For any fixed value of p, with scaling slope of $\log p$, the expected first passage time can vary over the interval $(1{,}1 + 1/(1-p))$. This example demonstrates that there is no necessary one-to-one relationship between scaling slope and expected first passage time, even for random walks on a fixed network structure and considering only variations in the stepping probabilities. Of course, both statistics—scaling slope and first-passage time—depend at least in part on the common parameter p. In that sense, the two are related. However, one can construct other network walks where scaling slope and expected first-passage time do not depend on any common parameters. We leave this as an exercise for the reader. Supplementary Figure 3B provides a similar example for powerlaw scaling, by modifying the walk in Supplementary Figure 2B. The q versus $1-q$ branches from the START node influence the expected first passage time, but not the slope of the powerlaw relationship between path probability and path probability rank.

Relationships between the path distribution and mixing times. In a similar vein, there is no necessary relationship between the scaling slope and the mixing time of a walk. Mixing time can be defined in different ways, but intuitively, it quantifies how quickly a walk on a network reaches a "steady-state" distribution. (In fact, a quasi-steady state distribution over the live nodes is perhaps a more relevant notion in our context, but this distinction is not important.) Consider, for instance, the memoryless random walk on N nodes, which we have discussed several times. This family of walks, parameterized by N, has a mixing time of 1 step regardless of N. This is because the relative probabilities of the different nodes at a given step are independent of the previous node. As discussed previously, however, the slope of the powerlaw scaling of this walk depends on N. Therefore, this family of random walks demonstrates that there need be no relationship between scaling slope and mixing time of a walk.

There is a different sense in which path distributions and mixing times are related, however. The reader may have noticed that in our plots of $\log P_r$ against either $r^{1/k}$ or $\log r$, the paths with lowest rank r are not always so consistent with the overall stretched exponential or powerlaw relationship. This is because the relationships we establish are asymptotic—they hold for sufficiently large ranks r. The question arises, then, as to what constitutes "sufficiently large" in any particular case. For simplicity, let us restrict attention to the multi-cyclic case, where $\log P_r / \log r \to b$, where b is the scaling slope. As we state in Supplementary Note 1, we can obtain the scaling relationship by looking at an "expanded network" (Supplementary Figure 1D), where each pseudo-transition has probability p. Roughly speaking, the approach to slope b depends on the mixing time of that expanded network. Slightly more formally, the probability of an n-step path from START to END is p^n, while the rank of such paths grows approximately as $e^{\lambda_1 n}$. This is why we have

$$\lim_{r\to\infty} \frac{\log P_r}{\log r} = \lim_{n\to\infty} \frac{\log p^n}{\log e^{\lambda_1 n}} = \lim_{n\to\infty} \frac{n \log p}{\lambda_1 n} = \frac{\log p}{\lambda_1} = b \ . \tag{3}$$

The fact that the number of paths grows approximately as $e^{\lambda_1 n}$ is well known from linear algebra / Frobenius-Perron theory [7]. The constant λ_1 is the modulus of the largest eigenvalue of the adjacency matrix of live nodes in the expanded graph. However, a slightly more detailed analysis would include both the largest and second largest eigenvalues. That is, the rank of the n-step paths can be bounded by $e^{\lambda_1 n}(1 \pm ce^{-\delta n})$, where $\delta = \lambda_1 - \lambda_2$ is the spectral gap, or difference

in modulus of the largest and second largest eigenvalues, and c is some constant. The spectral gap characterizes the mixing time, and controls how quickly the number of paths converges to its largest-eigenvalue exponential. Thus, when analyzing the path distribution, the rate of approach to the limiting relationship between $\log P_r$ and $\log r$ can be analyzed in terms of the mixing time of the expanded graph. This is not the same as the mixing time of the original walk. However, it establishes that there is some relationship between the path distribution and a mixing time that is related to the original random walk on the network.

Supplementary Note 4: Autocorrelation and cross-validation analysis of history dependence in note sequences

As stated in the main text, we employed K-order random walk models to analyze note sequences in songs from the Essen database. We found that the best match between predicted scaling slope and observed scaling slope was achieved for $K = 5$, suggesting that a 5-step history dependence was best at capturing correlations in the note sequences. It appears that shorter history dependence is insufficient. Longer history dependence, of course, should also allow such correlations to be captured. However, the longer the history dependence gets, the more the model is in danger of overfitting the data. To further test whether $K = 5$ is a good choice, we employed two other accepted methods for assessing history dependence in symbolic (i.e., non-numeric) sequences.

First, we conducted an autocorrelation analysis of the songs using the "equal-symbol autocorrelation" approach of Voss [8]. In the present context, the autocorrelation at lag τ is the fraction of times, over all songs, that a note or rest is the same as the note or rest that comes τ steps before. The autocorrelations for τ up to 10 are listed in the table below, along with similar autocorrelations computed from randomly permuted songs, and Bonferonni-corrected p-values (by unpaired t-test) for significance of difference between the two. The autocorrelations are also visualized in Supplementary Figure 4, along with 95% confidence intervals.

Lag τ	Autocorrelation songs	Autocorrelation permuted	p-value
1	0.1865297039	0.1795900345	1.1149e-16
2	0.2008514963	0.1805937642	3.2290e-130
3	0.1660867442	0.1789347833	4.6183e-56
4	0.1758773042	0.1795533386	8.2761e-05
5	0.1685246057	0.1800946967	1.5815e-43
6	0.1737748832	0.1807575585	9.7524e-16
7	0.1775437486	0.1806890481	0.0023
8	0.1823804701	0.1804483933	0.2569
9	0.1785760371	0.1788134675	1
10	0.1802579297	0.1797144199	1

The strongest deviations between correlations in the real songs versus the permuted songs are seen at time lags of $\tau = 2, 3$ and 5, with statistically significant, though weaker, deviations up to $\tau = 7$. This suggests an upper limit of 7 on the appropriate degree of history dependence, with a shorter history dependence possible. (Typically, with increasing τ the autocorrelation function "decays" towards a neutral value, rather than jumping abruptly to a neutral value. Intuively, this happens because if the symbol at position i is related to the symbol at position $i - \tau$, then by transitivity, it is also related to the symbol at $i - 2\tau$—though the relationship is likely weaker.) In any case, the upper limit of $\tau = 7$ is broadly consistent with our scaling-based estimate of length-5 history dependence. If one interprets the significant difference in real and permuted autocorrelations at $\tau = 5$ as substantial, and the lesser autocorrelations at $\tau = 6$ and 7 as "decay" towards the neutral value, then the autocorrelation analysis is completely consistent with our scaling-based analysis.

We also tried another approach to estimating the appropriate degree of history dependence. In this approach, we randomly divided the set of songs into two distinct subsets, a "training" set and a "testing" set. Each set had 4233 songs. We then used the training set to compute the maximum a posteriori estimate of a K-order Markov model, under the assumption of uniform

Dirichlet priors. In short, if there are N possible K-tuples, and if a particular K-tuple x is at the start of M songs, then we estimate the probability of transition from the START node to K-tuple x as $(M+1)/(4233+N)$. (The maximum likelihood estimate would be just $M/4233$, but this has the well-known problem of estimating zero probability for some initial K-tuples, so that the testing set has zero apparent probability under the estimated model.) Then, to estimate the probabilities of different K-tuple transitions during the songs, including a transition to an END node, if K-tuple x occurs M times, and is followed by K-tuple y on P of those times, and if there are $S = 14$ distinct possible symbols (i.e., notes, rests, and an "end node" marker, comprising all possible next node transitions), we estimate the probability of the transition for x to y as $(P + 1)/(M + S)$. Once all the transitions of the K-order Markov chain model are estimated, we evaluate the log probability of the songs observed in the testing set. For varying K, the results are shown below.

K	Training set log-prob	Test set log-prob
1	-190911.3645	-193753.7715
2	-180819.3848	-184600.6370
3	-174734.8906	-181247.1159
4	-172440.8256	-185827.6412
5	-174686.3545	-200518.3329

As is typical in scenarios like this, as the model gets more complicated (i.e., larger K = more parameters), the fit to both training and testing data initially improves. At some point, however, the model starts overfitting the data. Specifically, the test set probability increases only up until $K = 3$, and afterwards declines, suggesting that the models with $K = 4$ and higher are overfitting the training set. This analysis thus suggests that a history length of no less than 3 is optimal – from a prediction standpoint, and keeping in mind that only half of the data is used in the model estimation process. We say no less than 3 because more data might reduce the overfitting problem, and lead to a larger estimate of the best K. The necessity of accounting for the many different K-tuples that might follow the START weighed very heavily on more complex models, limiting K.

In the end, then, it appears that our scaling-based estimate of the degree of history dependence (5 steps) is right in the middle of a cross-validation-based estimate (at least 3 steps) and an autocorrelation-based estimate (at most 7 steps), lending some credence to the approach.

Supplementary Note 5: Powerlaw distributions for exit paths from energy basins of the G-protein model

As stated in the main text, by performing cut-based free energy analysis of the Scalco and Caflisch G-protein model [9], we divided the network into 7 major energy basins. Within each basin, the node with highest steady state probability was designated the START state, and all transitions out of a basin were routed to a new, generic END state. For every basin, the exact probabilities of the 10,000 most-probable exit paths are strongly consistent with a powerlaw path distribution (see Supplementary Figure 5), with slopes close to those predicted by our theory.

References

[1] R. Edwards, E. Foxall, and T. J. Perkins. Scaling properties of paths on graphs. *Electronic Journal of Linear Algebra*, 23:966–988, 2012.

[2] B. B. Mandelbrot. On recurrent noise limited coding. In E. Weber, editor, *Information Networks, the Brooklyn Polytechnic Institute Symposium*, pages 205–221, 1955.

[3] G. A. Miller. Some effects of intermittent silence. *The American Journal of Psychology*, 70(2):311–314, 1957.

[4] W. Li. Random texts exhibit Zipf's-law-like word frequency distribution. *IEEE Transactions on Information Theory*, 38(6):1842–1845, 1992.

[5] J. A. Bondy and U. S. R. Murty. *Graph theory with applications.* North-Holland, New York City, 1976.

[6] F. Roberts and B. Tesman. *Applied combinatorics.* Chapman and Hall/CRC, 2011.

[7] A. Berman and R. J. Plemmons. *Nonnegative matrices in the mathematical sciences.* Society for Industrial Mathematics, 1994.

[8] R. F. Voss. Evolution of long-range fractal correlation and 1/f noise in DNA base sequences. *Physical Review Letters*, 68(25):3805–3808, 1992.

[9] R. Scalco and A. Caflisch. Equilibrium distribution from distributed computing (simulations of protein folding). *The Journal of Physical Chemistry B*, 2011.
